# Advance in strategies to build efficient vaccines against tuberculosis

**DOI:** 10.3389/fvets.2022.955204

**Published:** 2022-11-24

**Authors:** Wei Qu, Yinhui Guo, Yan Xu, Jie Zhang, Zongchao Wang, Chaoyue Ding, Yuanhu Pan

**Affiliations:** ^1^National Reference Laboratory of Veterinary Drug Residues, MOA Key Laboratory for Detection of Veterinary Drug Residues, Huazhong Agricultural University, Wuhan, China; ^2^MOA Laboratory for Risk Assessment of Quality and Safety of Livestock and Poultry Products, Huazhong Agricultural University, Wuhan, China

**Keywords:** tuberculosis, immunization, vaccine, construction strategy, bioinformatics tools

## Abstract

Tuberculosis is a chronic consumptive infectious disease, which can cause great damage to human and animal health all over the world. The emergence of multi-drug resistant strains, the unstable protective effect of Bacillus Calmette-Guérin (BCG) vaccine on adults, and the mixed infection with HIV all warn people to exploit new approaches for conquering tuberculosis. At present, there has been significant progress in developing tuberculosis vaccines, such as improved BCG vaccine, subunit vaccine, DNA vaccine, live attenuated vaccine and inactivated vaccine. Among these candidate vaccines, there are some promising vaccines to improve or replace BCG vaccine effect. Meanwhile, the application of adjuvants, prime-boost strategy, immunoinformatic tools and targeting components have been studied concentratedly, and verified as valid means of raising the efficiency of tuberculosis vaccines as well. In this paper, the latest advance in tuberculosis vaccines in recent years is reviewed to provide reliable information for future tuberculosis prevention and treatment.

## Highlights

- Although BCG vaccine is the only vaccine approved for tuberculosis prevention, its protective efficacy still needs further improvement.
- Multivalent subunit vaccines and DNA vaccines with good safety and targeting ability have the potential to replace BCG vaccines.
- Live attenuated vaccines are effective against tuberculosis, and the key lies in reliable targeted mutation technology.

## Introduction

Tuberculosis is a chronic consumptive infectious disease caused by Mycobacterium tuberculosis (Mtb), which is greatly harmful to humans and animals (1–4). According to World Health Organization Global Tuberculosis Report, 10 million people were infected with tuberculosis and 3.44 million people died from tuberculosis in 2020 (5). Although human tuberculosis is a surmountable disease currently, the course of treatment is a long-term process, which can be influenced by many factors, such as medication continuity, patient compliance and clinical intervention timing. In addition, as the only available vaccine against tuberculosis, BCG's preventive effect is still limited and unstable yet (6, 7). On the other hand, diverse domestic and wild animals are potential hosts of Mycobacterium bovis (Mb), which may threaten the prevention and control of human tuberculosis. Furthermore, besides various wildlife reservoirs (brushtail possums, badgers, ferrets, elks, etc.) of Mb, it is estimated that the cost of national bovine tuberculosis control programme will exceed 1 billion pounds in the UK during 2014–2024 (8). And the cost of bovine tuberculosis reaches 3 billion dollars a year globally (4). Apart from a few countries, which can control bovine tuberculosis by test-and-slaughter strategies, it is hard to reduce the prevalence of bovine tuberculosis for other countries (especially for developing countries). Therefore, it is an urgent priority to seek efficient approaches for the prevention and control of this dangerous and persistent zoonosis (3).

In the *in vitro* treatment trial of tuberculosis, some classic first-line drugs including pyrazinamide, rifampicin, isoniazid and ethambutol have shown satisfactory curative efficacy. However, in the actual therapies of tuberculosis, the structural characteristics of tubercle bacillus and the generation of drug-resistant strains have brought great obstacles to cure illness. On the one hand, Mtb cell wall is rich in lipids. A large number of mycolic acid molecules surround the peptidoglycan layer, and the outer layer mainly consists of long-chain fatty acids, making Mtb impermeable to most antibiotics (9). The drug resistance mechanism of tuberculosis was discussed in more details in another review (10). On the other hand, some drug-resistant genes of Mtb have been detected by DNA extraction and PCR (11). In 2020, 132,222 of the bacteriologically confirmed tuberculosis cases were multidrug-resistant or rifampicin-resistant tuberculosis. The number of tuberculosis cases resistant to rifampicin and any fluoroquinolone, and resistant to at least one drug including rifampicin, fluoroquinolone, bedaquiline and linezolid reached 25,681 (5). Remarkably, the drug resistance rates are keep rising. To protect against tuberculosis, BCG vaccine serves as the only effective product. Although it has protective effects on extrapulmonary tuberculosis in infants, its prevention efficacy on adults is very unstable (12). Moreover, the tricky part is that the combined infection of tuberculosis with Acquired Immune Deficiency Syndrome (AIDS) causes latent tuberculosis to become active tuberculosis, which has greatly increased the incidence and mortality of tuberculosis (6). Therefore, there is no delay in developing new tuberculosis vaccines which can either enhance BCG vaccine efficacy or stimulate stronger immune effects by means of alternative approaches. According to previous studies, this review summarizes the latest advances of new tuberculosis vaccines in the last decade, including BCG vaccine, evolutionary products that improve existing BCG vaccine performance, novel substitutes such as subunit vaccines, DNA vaccines, and so on.

## Immune response to Mtb infection

**Figure 1:**
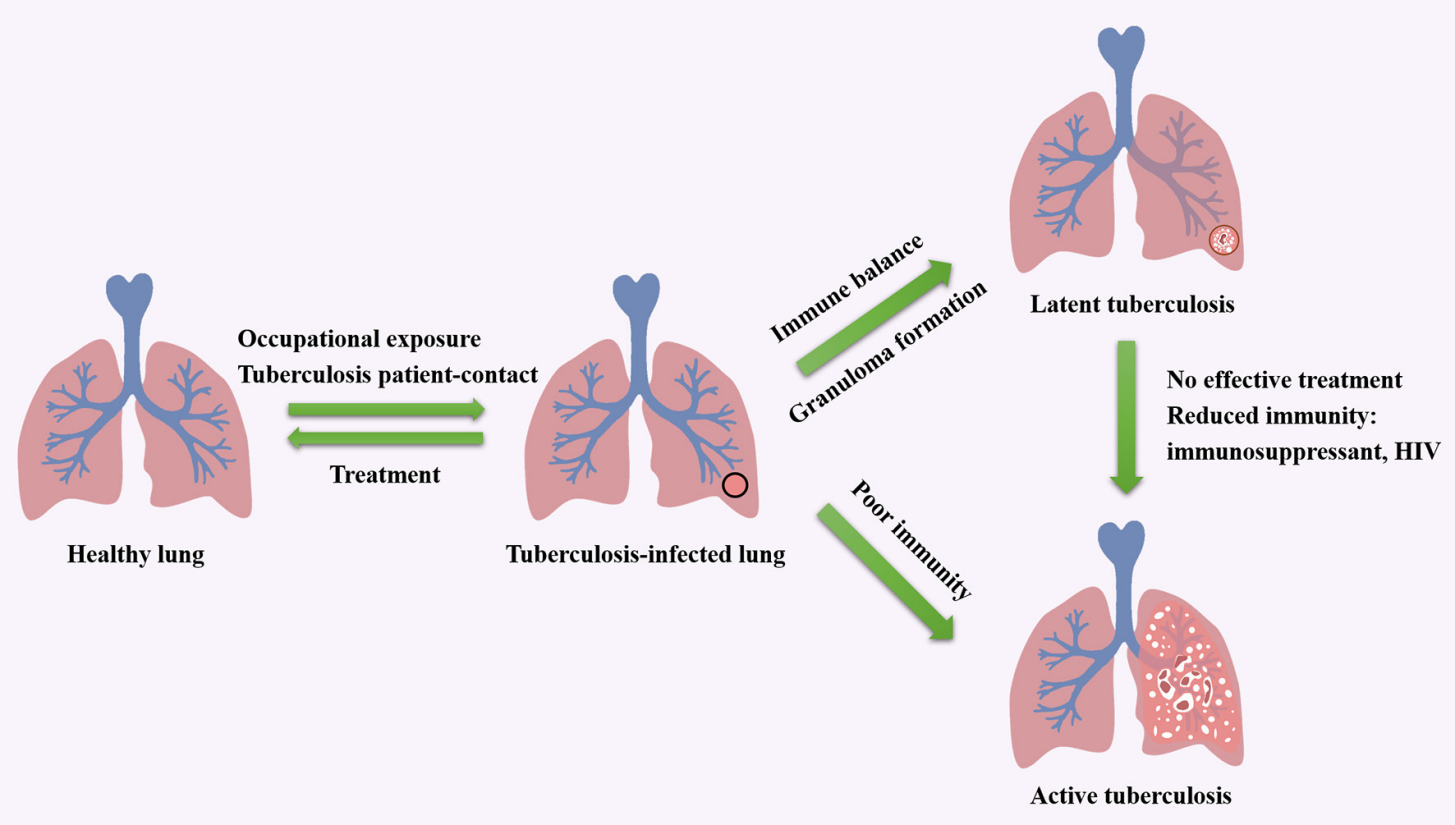
The cycle of tuberculosis infection and the factors that impact the development of tuberculosis.

As an airborne zoonosis, tuberculosis is mainly transmitted by aerosol. If Mtb reach deep alveoli, they will be swallowed by alveolar macrophages and interstitial dendritic cells. The pathogen can lie dormant in macrophages without any replication, which leads to latent infection. And latent infection develops into active disease in asymptomatic or potential infected people and animals after immune system suppression. The factors influence the course of tuberculosis infection including host, bacteria and treatment. Tuberculosis patient-contact, health status, daily habits, vaccination, age and immunity are related inducements for hosts (12–14). The virulence, invasiveness and drug resistance of Mtb also affect the process of infection. Moreover, by producing phosphatase, serine/threonine kinase, Mtb can inhibit the maturation of phagosomes formed after being engulfed by macrophages (15, 16). Meanwhile, Mtb escapes from the body's immune system by expressing anti-stress genes, inhibiting autophagy and inactivating reactive oxygen species, and forms granulomas and causes latent infection (17–19). The influencing factors in treatment include medical conditions, economic status, patient compliance. The cycle of tuberculosis infection and the factors that impact the development of tuberculosis are shown in Figure 1. Additionally, alveolar macrophages are the main invasion targets of Mtb. In infected cells, not only the fusion of phagosomes and lysosomes is blocked, the production of reactive nitrogen intermediates (RNIs) is also hindered (7, 20, 21). This enables bacteria to tolerate the intracellular environment. In this progress, the activation of macrophages is of great importance. Medley et al. (22) discovered potential vaccine and drug targets in the interaction of Mtb and macrophages, such as the enzymes encoded by Cytochrome P450 125 gene and Cytochrome P450 142 gene, which involved in the cholesterol metabolism of Mtb.

**Figure 2:**
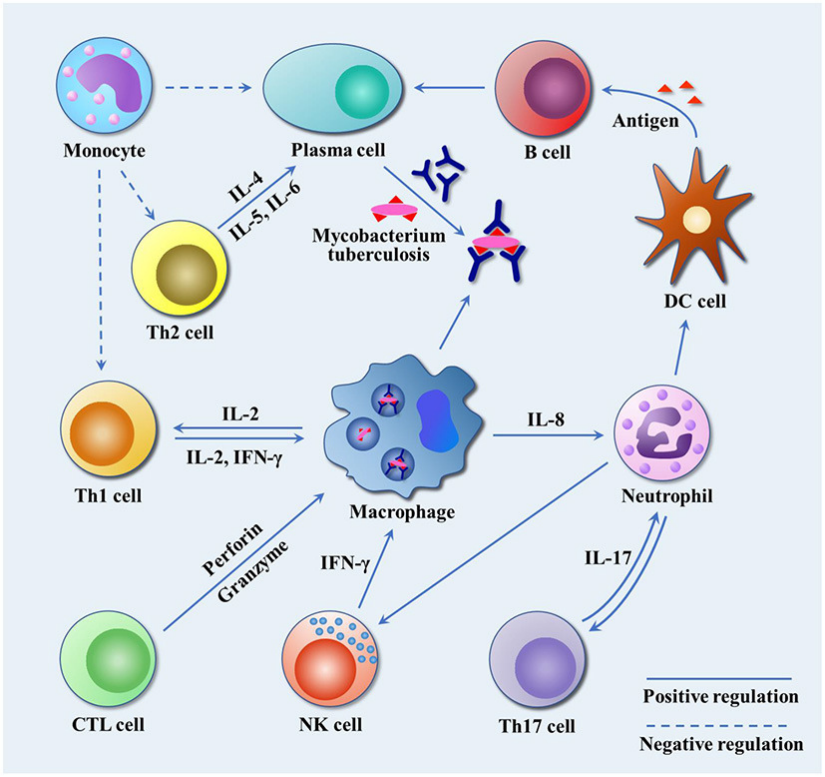
The immune response to Mtb infection. Mtb travels through the respiratory tract to the lungs, where it is engulfed by alveolar macrophages. By inhibiting the fusion of phagosomes and lysosomes, and blocking the production of active nitrogen intermediates, it escapes the body's immune killing and cause latent infection. Infected macrophages release IL-8 to attract neutrophils to the site of infection, and neutrophils in turn release chemokines to attract dendritic cells, NK cells and other immune cells to the site of infection to play an anti-tuberculosis role. IL-17 secreted by Th17 cells promote airway epithelial cells to produce IL-8 and G-CSF to attract more neutrophils, and participate in T cell-mediated IFN-γ and granuloma formation. NK cells release IFN-γ to promote Th1-type immune response. Dendritic cells, as professional antigen presenting cells, present Mtb antigen for B cells. B cells release antigen-specific antibodies to kill extracellular Mtb or Mtb released after macrophage death. Th2 cells release IL-4, IL-5 and IL-6 to assist the activation of B cells and promote the production of specific antibodies. In addition, macrophages release IL-2 to promote the proliferation and differentiation of Th1 cells, and Th1 cells secrete IL-2 and IFN-γ to reactivate infected macrophages. CTL cells differentiated from CD8^+^ T cells release perforin and granzyme to kill tuberculosis infected cells. Monocytes inhibit Th1 and Th2 by releasing TGF-β, and suppress the production of tuberculosis antibodies.

**Figure 3:**
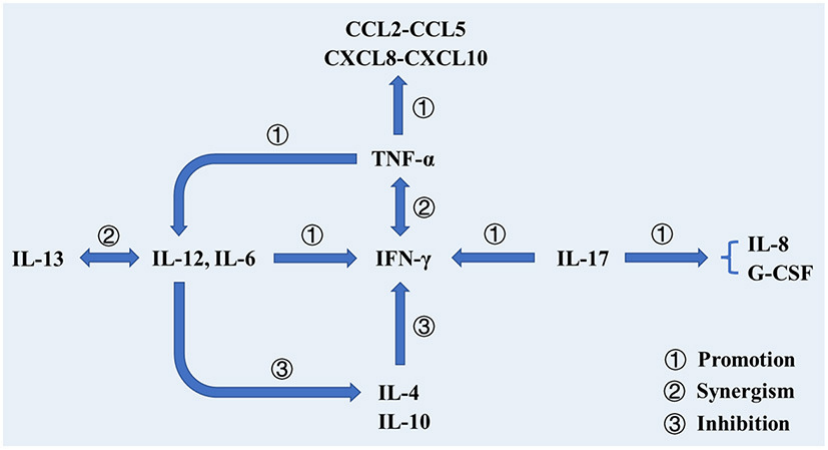
The roles of various biomarkers involved in the protection against tuberculosis. In addition to cooperating with IFN-γ to activate macrophages to phagocytose mycobacterium tuberculosis, TNF-α also activates T cells, prompting them to produce cytokines. Additionally, TNF-α can promote DC differentiation and promote IL-12 production. Il-12 cooperates with IL-13 to promote the production and efficacy of IFN-γ and inhibit the production of IL-4. IL-17 participates in the production of IFN-γ and promotes the production of IL-8 and G-CSF, thereby stimulating neutrophils to the site of infection.

After infected by Mtb, a series of immunologic cascade reactions are initiated. Mononuclear macrophages release TNF-α to activate T cells. Meanwhile, T cells divide into CD4^+^ T cells and CD8^+^ T cells after the stimulation of tuberculosis antigen. CD4^+^ T cells differentiate into Th2 cells, and IL-4, IL-5, and IL-6 secreted by Th2 cells can assist the activation of B cells to promote the production of antibodies against Mtb. IL-2 and IFN-γ produced by Th1 cells participate in the activation of macrophages together with the aforementioned cytokines (TNF-α, TNF-β), and facilitate the development of Th1-type immune responses. These cytokines induce anti-mycobacterial activities by activating macrophages, forming granulomas, increasing antigen presentation or producing effector molecules, respectively (2). As a biomarker of Th2-type immune response, excessive IL-4 has an antagonistic effect on the recovery of tuberculosis infection (23, 24). Buccheri et al. (25) confirmed that IL-4 deficiency could increase the host's resistance to tuberculosis infection. Monocyte macrophages and Th2 cells also play regulatory roles, which produce inhibitory transforming growth factor-β (TGF-β) and IL-10, respectively. They can prevent the activation of T lymphocytes by inhibiting the production of certain cytokines, e.g., IFN-γ, TNF-α and IL-12. There have been convincing evidences to prove that tuberculosis can induce IL-10 and TGF-β to suppress immune responses (21, 26, 27). As illustrated in Figure 2, cellular immunity and related cytokines play an important role in anti-tuberculosis infection, which is also essential to the protection potency for BCG vaccine. In addition, the roles of various biomarkers involved in the protection against tuberculosis are shown in Figure 3. As a result, the vaccines tend to induce Th1-type immune responses are preferred. However, it does not mean that Th2-type immunity and humoral immunity play an accomplice role of tuberculosis (28, 29). And this implies that it is necessary to seek the balance between cellular immunity and humoral immunity for improving a more effective tuberculosis vaccine.

## BCG vaccines

BCG vaccine is a live attenuated vaccine made from suspensions of attenuated bovine type tubercle bacilli with enhanced macrophage activity, which potentiates the ability of macrophages to eliminate intracellular parasitic bacteria. However, as mentioned above, the protection efficacy of this classic vaccine is variable in adults, which limits its application. Some researchers put forward that the reason for this change might be the effect of environmental mycobacteria. It masks or blocks the efficacy of BCG vaccine (28). The masking hypothesis supposes that the immunity caused by environmental tuberculosis hides the additional effect of BCG vaccine. According to the blocking hypothesis, the immunity caused by environmental tuberculosis eliminates BCG vaccine and prevents BCG vaccine from working. Apart from environmental mycobacteria, the effector memory T cells stimulated by BCG vaccine will attenuate with time, which is crucial to the protection against tuberculosis (30, 31). The nutritional status of host is crucial to maintain the good reaction capacity of immune system, which accounts for organic resistance to infections, including tuberculosis. And the malnutrition may influence prospective effects of BCG vaccine by inhibiting the production of T cells and some cytokines (32). To improve the efficacy of BCG vaccine, some adjuvants (lipopeptide, fusion protein and lactoferrin) were used and achieved ideal experimental results (33–35). Moreover, researchers proposed that the optimal activation of CD4^+^ and CD8^+^ T cells was significant, and the inability of BCG vaccine to effectively activate CD8^+^ T cells was the reason for its poor performance (29).

On the other hand, alveolar lining fluid (ALF) is an internal factor that may impede the function of BCG vaccine. In accordance with the route of natural infection of Mtb, the first barrier lying in respiratory tract is ALF. ALF is composed of surfactant lipids and associated proteins, as well as of an aqueous hypophysis rich in innate host defense molecules, including hydrolytic enzymes, surfactant proteins A and D, complement proteins and immunoglobulins (36). Under natural conditions, hydrolases remove some lipids, such as dimethyl trehalose at the periphery of the cell wall of Mtb (37). The new antigen motif will be exposed, which is bound to affect the immune responses caused by Mtb *in vivo* (36). These changes may affect the uptake and phagocytosis of Mtb by immune cells. BCG vaccine is usually injected intracutaneously without ALF treatment, and the *in vivo* protective effects cannot reverse this blocking influence. Accordingly, what is noteworthy is that the potency of BCG vaccine can be improved by inoculating mice with ALF-treated BCG vaccine (36).

As the first antituberculosis vaccine, BCG has been applied to clinical use over 100 years. During this time, continuing passage of BCG strains have produced various substrains with different genetic mutations, such as BCG Pasteur, BCG Danish, BCG Sweden, and so on (38). The diverse immune efficacy of these substrains within animal models were described by numerous studies and systematically summarized in a previous review (39–42). A random trial carried out in Hong Kong involved 303,092 infants revealed that BCG Pasteur vaccine could reduce the tuberculosis risk by 45% in comparison with that of BCG Glaxo (43). And there was a similar phenomenon observed in a Kazakhstan study of the tuberculosis risk related to neonates, resulting in a reduction of 69%, 22% for BCG Tokyo and BCG Moscow vaccination, respectively (44). However, these seems to be unconvincing evidences owing to distinct study protocols, for instance, inoculation regimen, administration route, challenge schedule and immune evaluation, which failed to attribute the various protection efficacy to BCG strains (39, 45, 46). Moreover, no significant influence was detected among different BCG strains through meta-analysis (45). And some studies suggested the potential reasons might lay in exposure to environmental mycobacteria, prior infection of Mtb and the environment condition of trials (45, 47–49). Therefore, it is hard to determine the relationship between BCG strains and immune efficacy, and a standard scheme including experiment operations and evaluating indicators is an urgent need for assessing vaccination potency of different BCG strains.

In addition, microtubule-associated protein 1A/1B-light chain 3 (LC3) related phagocytosis and autophagy enhance antigen presentation and may contribute to vaccine efficacy, while Mtb can inhibit above effects. And BCG vaccine strain elicits even less LC3-trafficking than Mtb, which may explain its limited efficacy (50). Köster et al. knocked out the CpsA gene which could inhibit LC3 traffic pathways from mc26206 vaccine strain (ΔCpsA) and inoculated mice. They found that vaccination of mice with mc26206, mc26206 ΔcpsA and BCG Danish resulted in similar protective efficacy against Mtb challenge (50). These phenomena suggest that the improvement of BCG vaccine in some aspects may be limited. Some genes missing in BCG vaccine can be supplemented by appropriate means for immune enhancement, but the impact of environmental tuberculosis is difficult to eliminate. However, as the only tuberculosis vaccine currently available, it is an excellent choice to use BCG vaccine as a breakthrough.

Another problem of BCG vaccine is that it brings doubts in the distinction between primary tuberculosis infection and vaccination both in human and animal. The tuberculin skin test is fundamental to diagnose tuberculosis. However, previous BCG vaccine inoculation may interfere with the tuberculin skin test results, which is one of the most important obstructions for animal vaccination against tuberculosis. Recently, Chandran et al. developed a modified BCG vaccine, which could distinguish between guinea pigs inoculated by BCG vaccine and infected with tuberculosis (51). In its preparation of BCG vaccine, some antigen (ESAT-6, CFP-10, etc.) genes were deleted from the genome. The specific antigens between BCG vaccine and environmental strains were used to stimulate the production of IFN-γ in peripheral blood mononuclear cells, which contributed to distinguish vaccinated animals from naturally infected animals. However, this method requires strict experimental conditions. They deleted unnecessary genes for the function of BCG vaccine. These genes, together with ESAT-6 and CFP-10, were used in a skin test for identification (51). The diagnostic and preventive efficacy of this modified BCG vaccine needs to be further validated. Nevertheless, this strategy holds great promise for the control and prevention of tuberculosis as well as other diseases.

Besides, the application of BCG vaccine is lethal for HIV patients with immunodeficiency (52). And TBVAC85, a derivative of mycobacterial shottsii expressing Mtb antigen 85B, is considered a safer alternative to BCG vaccine. Inoculated guinea pigs were subsequently challenged with tuberculosis aerosols and it was found that the protective effect of TBVAC85 was similar to that of BCG vaccine. Moreover, it has a higher safety profile than that of BCG vaccine and deserves further study (53). It is expected to provide a reference regimen for tuberculosis prevention in HIV patients. Moreover, Muttaqin et al. fabricated a combined vaccine of tuberculosis and AIDS, and screened out the dominant epitopes of the two through immune epitope database. In theory, the MHC-I and MHC-II epitope coverage of this vaccine can reach 85 and 99% around the world, respectively. To verify its *in vivo* effect, further animal experiments need to be conducted (54).

BCG vaccine is injected intradermally nowadays, but its original administration route is oral inoculation. And it is still a challenge to evaluate diverse vaccination routes for the judgement of best delivery approach to stimulate superior protective immune effects. To protect from pulmonary tuberculosis, aerosol might be a better way to delivery BCG vaccine, which can induce the mucosal immunity within trachea (55). Meanwhile, in comparison with transdermal delivery routes, intranasal or inhalation taken BCG vaccine stimulated more effective protective immunity (56–58). And aerosol vaccination is a promising method to maximize the immune effect of BCG vaccine. In addition, intravenous injection of BCG vaccine is proved to be more effective than other routes in animal model. In Rhesus macaques, intravenous inoculation of BCG vaccine exhibited a better protection without pulmonary lesion than that of intradermal inoculation (59, 60). Nevertheless, prior to efficacy, its intravenous application in human needs comprehensive examinations to ensure safety.

Despite above defects, there have been some attempts to explore the protection effect of BCG vaccine against animal tuberculosis recently. To assist the control of bovine tuberculosis for the British cattle, an evaluation was performed by inoculation with BCG Danish in neonatal calves. Two independent groups of cattle were endotracheally challenged by Mb after 12 and 24 months postvaccination, respectively. By comparison with the unvaccinated group, the lung and lymph node pathology scores reduced in both vaccinated groups, while the reduction of the 12 months group was significant statistically. The antigen-specific IFN-γ remained in vaccinated cattle's blood for about 90 weeks. The IFN-γ-secreting central memory T cells of the 12 months group were obviously abundant than those of the unvaccinated group, while the difference between the 24 months group and the unvaccinated group was not notable. These observations proved the protection of BCG vaccine maintaining 12 months in cattle, and revaccination or boost programme might be needed to guarantee a long-term inoculation effect against Mb infection (61). For defensing natural Mb infection within free-living badgers, a field trial of oral vaccination of BCG vaccine was carried out. The median time to seroconversion of the vaccinated badgers was 413 days, which remarkably longer (p = 0.04) than 230 days of the unvaccinated badgers. The Vaccine Efficacy (VE) based on hazard rate ratios rose with the increase of vaccination rate. After the trail, a notable diversity of lesion proportion (9% for the vaccinated group, and 26% for the unvaccinated group) according to the Mb cultures from badgers was determined by post-mortem. And the results provided an alternative tool to control the tuberculosis incidence of wild badgers (62). Moreover, other studies have demonstrated the protection effects of oral BCG vaccine strains Pasteur and Danish are similar in badgers (63). Recent anti-Mb studies concerning domestic animals and wild animals by means of BCG vaccine showed encouraging outcomes, which implied the feasibility of finding a suitable vaccine. Although there is absent of available vaccine for animal tuberculosis, it is still worth to keep investigating the practicability, inoculation strategy and immune reaction on the basis of BCG vaccine for developing new vaccines not only for animal but also for human.

## Improved BCG vaccines

Although the protective efficacy of BCG vaccine is unstable, it is the only prophylactic vaccine approved for use. Modification of BCG vaccine to improve its antituberculosis ability represents a relatively easy way to control tuberculosis. Studies in this field have also made promising results, for instance, a boost of immune competence through the addition of adjuvants and prime-boost strategies.

### Application of prime-boost strategy

The prime-boost strategy is usually to inoculate BCG vaccine first and then enhance it with homologous or heterologous vaccine. And other vaccines can be used for prime immunization as well (64). To evaluate the putative correlation between protective efficacy and CD4^+^ T cell subtype phenotype, Choi et al. constructed the HSP90-E6/CIA05 vaccine composed of HSP90 and ESAT-6 proteins accompanying with adjuvant CIA05. By applying a prime-boost strategy in combination with BCG vaccine, the results demonstrated that mice in the HSP90-E6/CIA05 boost group exhibited significantly less lung inflammation and lesion areas compared with those in the BCG group and the ESAT-6/CIA05 enhanced group. Moreover, the bacterial burden in the lung and spleen was significantly lower in HSP90-E6/CIA05-boosted mice than that in ESAT-6/CIA05-boosted mice (65). CIA05 is a toll-like receptor 4 (TLR4) agonist purified from an Escherichia coli strain that expresses lipopolysaccharide (LPS) with short carbohydrate chains and detoxifies by alkaline hydrolysis (66). The ability of CIA05 to promote Th1-type immune response was proved in the development of human papillomavirus vaccine (67). These studies manifested the important role of TLR4 in anti-tuberculosis immunity (68). TLR4 also can activate MyD88-, MAPK- and NF-κB-dependent signaling pathways. The subunit vaccine composed of tuberculosis heat shock protein 90 family Rv2299c and ESAT-6 was developed based on this principle, which significantly improved the effect of BCG vaccine (69). Additionally, several tuberculosis antigens (HspX, PPE44 and EsxV) were encapsulated in liposomes containing dimethyldioctadecylammonium/trehalose-6,6-dibehenate as a boost vaccine, which was used after BCG vaccine. In terms of the generation of related cytokines (IFN-c, IL-12 et al.), IgG and enhancement of immune memory after vaccination, this vaccine exhibited a strong Th1 promoting ability along with a prominent BCG vaccine enhancing effect (70). And more encouraging results have been reported by application of prime-boost strategy (33, 69, 71–73). The combination of primer and booster mainly depends on the BCG potency. The common components of primer or booster other than BCG vaccine in prime-boost strategy include antigen, lipopeptide and live attenuated strain, which promote the cellular immune responses caused by BCG vaccine. And this strategy maybe meaningful to improve the immunogenicity of attenuated BCG strains, and in turn to distinguish critical components in BCG vaccine for keeping its protection potency.

Moreover, the constituent antigens of boost adjuvants are worth to design elaborately. Researchers found that the DNA vaccine expressing tuberculosis antigen Ag85a had a protective effect on mice when used for prime immune, while its protective effect disappeared when used for boost immune (74). A phenomenon called original antigenic sin may be able to explain it well. The original antigenic sin suggests that *in vivo* immune system makes an immune response and forms immune memory based on the initial antigen immunity. When the same antigen with slight changes in its epitope appears again, immune system will follow existed immune memory instead of adapting to its slight differences of the epitope (75). This phenomenon is attributed to the cross-reaction of T helper cells and antigens (76). Therefore, in order to achieve desired prime-boost strategy, the immune responses triggered by the selected antigen of boost adjuvants should consistent with original T cell responses introduced by the initial immunization of BCG vaccine.

The inoculation interval of prime-boost strategy is also worth investigating. MTBVAC, obtained from live attenuated Mtb strain, was constructed under the genetic background of clinical isolate MT103. Its two genes, PhoP and fadd26, are necessary for the virulence of Mtb (77, 78). The evaluation of MTBVAC showed a better immune potency by applying prime-boost strategy. Two vaccinations with an interval of either 6 or 20 weeks exhibited an enhancement effect in the BCG prime-MTBVAC boost group. For the MTBVAC prime-BCG boost group, the results of two inoculations with an interval of 6 weeks indicated a similar facilitation effect. However, with the extension of vaccination interval, its protective effect gradually decreased to the level of the BCG group (79). The study of GamTBvac vaccine by Vasina et al. was also concerned with inoculation intervals. After a long-term BCG vaccination, GamTBvac could still improve its effect. GamTBvac, a new candidate vaccine as a BCG vaccine booster, contains dextran binding domain modified Ag85A and ESAT6-CFP10-Mtb antigens and CpG ODN adjuvant. It was proved that the semi-dose vaccine group had stable IFN-γ production ability and higher antigen-specific IgG titer compared with the low-dose and high-dose vaccine groups (80). Until now, a universal inoculation interval of prime-boost scheme has not been given by existing researches. Different kinds and sequences of primer and booster often generate various protection effects. This may be attributed to the lack of complete recognition in tuberculosis immunity and related key points of protection. However, it is necessary to build an operation standard including dosage regimen, vaccination route, inoculation interval, animal model and evaluation indicator to provide reference for subsequent studies.

Additionally, some studies have shown that mucosal delivery enhances the generation of tissue-resident memory T cells, and these T cells may inhibit the early invasion of bacterial infection (81). Hence, it is rational to develop modified BCG vaccine administrated through mucosa in virtue of prime-boost strategy. Besides, Lactobacillus plantarum was utilized as a platform to produce tuberculosis antigen protein for BCG vaccine preparation, which had a certain protective effect (82). But it was not as fine as single parenteral inoculation of BCG vaccine (83).

Although it has been absent of approved vaccines for animals so far, constructive efforts including prime-boost strategy have been conducted to make full use of BCG. A long-term evaluation over 2 years tested the protection effect of revaccination with three different boost agents (BCG, ESAT-6 and Antigen 85A complexes, Mb culture filtrate) in BCG-vaccinated cattle. After endotracheally challenged with virulent Mb, cattle were sacrificed and necropsied. The lung and pulmonary lymph node lesion scores were apparently reduced in above three prime-boost groups in contrast to the unvaccinated group, while a single inoculation with BCG vaccine could not protect cattle from Mb challenge (84). In addition, immunization boost by a human type 5 (Ad5)-based Ad5-Ag85A vaccine, Ad5-Ag85A/Rv0251/Rv0287/Rv0288 (Ad5-TBF) vaccine and protein TBF in emulsion adjuvant (Adj-TBF) vaccine in cattle was studied, which resulted in similar strengthen effects of the BCG-priming vaccination (85). These phenomena revealed revaccination could boost the protection of BCG vaccine in cattle. Meanwhile, oral bating is a feasible approach for vaccine administration in the wild, which is convenient for prime-boost strategy. Gortazar et al. (86) found that oral revaccination with BCG vaccine in Eurasian wild boar elicited a potent protection against Mb field strains under experimental conditions. These observations indicated revaccination of BCG vaccine is worth to extend to the future field vaccination trials for verifying its priority of prime-boost strategy.

### Introduction of adjuvants

Adjuvants are kinds of non-specific immune enhancers, which can facilitate the immune response to antigens or change the type of immunoreaction when administrated alone in advance or together with antigens. An appropriate adjuvant is an excellent supplementary for vaccines with low immunogenicity (87). The adjuvants summarized in this article are shown in Table 1. Combined with immune mechanism of tuberculosis, the application of adjuvants in autophagy, Th1-type immune response, cytokines and other aspects to improve the effect of BCG vaccine have been discussed subsequently.

**Table 1:** Summary of adjuvants applied in tuberculosis vaccines.

**Adjuvant**	**Mode of action**	**Validation animal (delivery route) and cell**	**References**
C5 peptide	Induce autophagy and activate TLR-2 pathway to exert anti-tuberculosis effect	C57Bl/6 mice (SC), primary bone marrow-derived MΦs(MΦs) and DCs from C57Bl/6 mice or primary bone marrow-derived MΦs (MΦs) and DCs from C57Bl/6 mice or TLR-2 KO mice	Khan et al. (88)
Dinucleotide SB 9200 (Inarigivir)	Participate in initiating an effective host immune response to intracellular infection	C57Bl/6 mice (SC), macrophages and DCs from C57Bl/6 mouse bone marrow	Khan et al. (89)
3,4,5,7-tetrahydroxyflavone	Inhibit potassium channel kv1.3 and promote the production of TCM	C57Bl/6 mice (IP), CD4^+^ T Cells	Singh et al. (77)
IFN-I	Promote the differentiation/activation of dendritic cells (DC) against specific antigens	BALB/c mice (SC), A549 cells, macrophages	Telesca et al. (90)
Alveolar lining fluid	Improve the effect of BCG	C57BL/6J or C3HeB, FeJ mice (SC), lung cells	Moliva et al. (36)
Deltamethrin Advax	Improve the immunogenicity of vaccine	Female C57BL/6 mice (IP), peripheral blood mononuclear cells	Counoupas et al. (91)
Retinoic acid (RA)	Regulate the differentiation of CD4^+^ T cells; Affect the homing of T cells and B cells; Affect the steady state of dendritic cells	Female CB6F1 mice (SC), bone marrow cells of mice	Riccomi et al. (92)
α-galactosylceramide	Promote the production of IFN-γ and IL-2	Female C57Bl/6 mice (IM), spleen and lung cells	Khan et al. (93)
Rv2299c	Activate dendritic cells	Female C57BL/6 mice (SC), murine bone marrow-derived DCs	Choi et al. (69)
Nanoemulsion	Induce strong mucosal IL-17t cell reaction	C57BL/6J mice (IM), lungs cells	Ahmed et al. (94)
CAF01	Promote antigen presentation and activate dendritic cells	Female CB6F1 (BALB/c × C57BL/6) hybrid mice (SC), isolated splenocytes	Thakur et al. (95)
FMS-like tyrosine kinase 3 ligands	Enhance DC recruitment and amplification and increase antigen uptake and cross-presentation	Female C57BL/6 mice (IM), glomerular mesangial cells	Ganzhu Feng et al. (96)
Chitosan	Protect plasmid from degradation; improve immunogenicity of vaccine; improve plasmid transfection efficiency	Female C57BL/6 mice (IM), glomerular mesangial cells	Bivas-Benita et al. (97)
IL-12	Give full play to their physiological activity	Cynomolgus monkeys, 15 K granulysin Tg mice, 3 types of 9 K granulysin Tg mice (IM), cells of lung, liver and spleen from mice	Yoko Kita et al. (98)
Microtubule-associated protein light chain 3	Enhance the reaction of CD4^+^ T cells by autophagy	C57BL/6 mice (SC), macrophages from mouse tibia and femur	Koster et al. (50)

Autophagy-facilitating and TLR-2 motivating C5 peptide originated from CFP-10 protein was over-expressed in BCG vaccine with Ag85B to prepare recombinant BCG 85C5 vaccine, in which C5 peptide and Ag85B acted as adjuvants. This vaccine not only promotes the production of Th1-type cytokines, but also inhibits MARCH 1 ubiquitin ligase which degrades MHC-II and enhances the surface expression of MHC-II in macrophages (88). Autophagy is involved in the immune mechanism against tuberculosis. And it is a beneficial attempt to improve autophagy-dependent interaction between antigen-presenting cells (APCs) and T cells in neonates and adults. Additionally, there is a critical review focused on constructing anti-Mtb vaccines with the help of autophagy targeting ability (99).

In addition to autophagy, Th1-type immune response plays an important part in anti-tuberculosis process. Although T-effector memory (TEM) cells, which generate Th1-type immune response cytokines (100), can effectively resist tuberculosis infection, they have low or no proliferation ability. On the other hand, T central memory (TCM) cells can generate TEM cells. Therefore, increasing TCM cells response and providing continuous TEM cells is a strong guarantee for long-term resistance to tuberculosis infection. Kv1.3 is a potassium channel, which is mainly expressed in TEM cells and crucial to the maintenance of related cells and the function of effectors. The knockdown of Kv1.3 led to the differential regulation between TCM cells and TEM cells, which was beneficial to the production of TCM cells (101). 3,4,5,7-tetrahydroxyflavone, also known as luteolin, a plant flavonoid that inhibits Kv1.3, has been studied. The effect of BCG vaccine was improved by giving luteolin to mice at the same time (102). Similarly, IFN-I (IFN-α and IFN-β) is important to the long-term survival of CD8^+^ T cells by promoting the differentiation/activation of human and mouse dendritic cells (DCs) in response to specific antigens (103–105). After took IFN-α and BCG vaccine in combination in mice, the immune results were superior to those of BCG vaccine, while single IFN-α treatment worsened pulmonary tuberculosis (90).

The dinucleotide SB 9200 (Inarigivir) is a nucleotide-binding oligomeric domain agonist containing protein 2 (NOD2) and retinoic acid-inducible gene I (RIG-I). Both are essential for initiating an effective host immune response against intracellular infection. Khan et al. mixed Inarigivir and BCG vaccine to inoculate mice, resulting in obvious growth of the expression level of IL-12, TNF-α, and IFN-β and relevant cytokines. It was also observed that the ability of macrophages to kill Mtb was improved and the bacterial load in the lungs of mice was reduced more significantly than that of BCG vaccine alone (89). Although the improvement effects of each kind of adjuvant are obvious, there remains many works to illuminate the correlations within immune mechanism of tuberculosis, and to seek most suitable adjuvants for promoting BCG vaccine.

## Inactivated vaccines

For preventing animal tuberculosis, inactivated vaccines are of great importance due to no diagnostic cross-reactions and rare virulent strain survival. Therefore, most of the reports focused on heat-inactivated Mb vaccines in recent investigations concerning animal tuberculosis prevention. Thomas et al. gave a challenge with Mb field strains to the red deer after their oral vaccination of heat-inactivated Mb (n = 5/group). In contrast to the unvaccinated control group, the tuberculosis lesion scores of the heat-inactivated Mb group showed a 53% reduction with significant differences. And the vaccination didn't interfere with the IFN-γ assay and antibody detection for TB diagnosis. The level of cytokines (TNF-α, IFN-γ, IL-1β, IL-10 and IL-12) in plasma kept steady after vaccination, while the challenge caused an increase of IL-1β level in all the groups (106). On the contrary, subcutaneous injection of BCG vaccine and intramuscular vaccination of heat-inactivated Mb elicited the response of IFN-γ and some antibodies, which hindered subsequent TB diagnosis (107, 108). As the immune mechanism of TB infection in red deer is still indefinable, the hint provided by the change of above cytokine levels is limited. And further study could make an improvement by increasing the number of animal samples for more representative data to reveal the immunologic profile.

As a primary natural host of Mtb complex in Mediterranean woods, the Eurasian wild boars confer a significant risk for the control of cattle tuberculosis. To evaluate the possibility of wild boar piglet vaccination against tuberculosis, a 4-year field trial was conducted in combination with a mathematical modeling analysis. The heat-inactivated Mb and BCG were filled into selective piglet feeders as vaccine baits in managed sites and natural sites. And the uptake ratio reached 50–74 and 89–92% in natural sites and managed sites, respectively. In comparison with local original tuberculosis morbidity (50–100%), the prevalence of the unvaccinated group increased 6%, while this number notably decreased by 34% in the heat-inactivated Mb group. And the differences in prevalence of other groups were not obvious. With the help of mathematical modeling, the long-term effect of this vaccination trial was assessed, which indicated the prevalence reduction of heat-inactivated Mb vaccinated piglets and the population size increasement of wild boars (109). Although this work had certain regional limitations, the above conclusions preliminarily demonstrated the application prospect of heat-inactivated Mb vaccines as a vital part of tuberculosis prevention for wild boars.

In another study concerning wild boars, the efficacy and safety of heat-inactivated Mb vaccine was investigated in a farm via parenteral inoculation. 3–4 months old piglets were inoculated by longissimus dorsi injection. They were revaccinated 1–2 months later and given yearly revaccination afterwards before their release or productive end. Observed by visual inspection or post-mortem test, there were no adverse reaction after vaccination. The prevalence of tuberculosis compatible lesion was 4.1 and 12.1% for the inoculation group and the control group, respectively. And the mean lesion score exhibited no difference (P > 0.05). The results indicated the safety and protection potency of this heat-inactivated vaccine for wild boar piglets against natural challenge. Moreover, this study implied the possible progress of heat-inactivated Mb in lesion prevalence decrease achieved by scheduled vaccination (110).

As goats are especially vulnerable to tuberculosis infection, a recent report estimates the protection effect of parenteral heat-inactivated Mb vaccine in goats. Mycobacterium caprae was applied to challenge the vaccinated (heat-inactivated Mb and BCG vaccine) and unvaccinated goats. Compared with the unvaccinated group, the relief of extrapulmonary and thoracic lesion volume, and less bacteria within pulmonary lymph nodes were observed in both BCG group and heat-inactivated Mb group with similar levels. Additionally, heat-inactivated Mb vaccine showed no influence on IFN-γ assay (111). These observations are in accordance with previous reports carried out in laboratory and field conditions (112, 113). Hence, to develop a safe and efficient heat-inactivated vaccine against tuberculosis for goats, more factors, such as dose delivery routes, lower challenge bacterial dosage and larger sample size should take into account to improve further assessments. Moreover, Mycobacterium caprae was utilized to build a tuberculosis infection model with gross lesions within the respiratory system in sheep for the first time. And heat-inactivated Mb vaccine and BCG vaccine were assessed in this model. As a result, the reduction of the bacterial load and lesion regions was remarkable in the BCG group, while the heat-inactivated Mb group showed no protection effect as regards to the bacterial load and lesion volumes. These different conclusions may be caused by the delivery route (oral vaccination for the heat-inactivated Mb group, subcutaneous vaccination for the BCG group) and the relatively low dose of the heat-inactivated vaccines, which is worth a further work to promote this heat-inactivated vaccine (114).

European badgers are considered to be a primary tuberculosis reservoir, which may be natural hosts to infect cattle. Based on the successful applications of heat-inactivated vaccines in red deer and wild boars, Balseiro et al. employed heat-inactivated Mb to protect badgers against tuberculosis. The vaccination was conducted by oral administration of heat-inactivated Mb or BCG, respectively. After endobronchial challenge with pathogenic Mb, the microscopic and gross lesions within tuberculosis-infected badgers of both vaccinated groups decreased in the degree and volume. The spread of Mb in vaccinated badgers was inhibited as well. And no adverse clinical signs were observed in the vaccinated groups. In endemic countries, this oral heat-inactivated vaccine was proved to be a potential candidate suit to badgers (115). Furthermore, in order to figure out the protection mechanism of heat-inactivated vaccines in badgers, the immune responses of lung granuloma cells were analyzed according to the lesion immunopathology. By inspection of the lung granulomas from vaccinated (heat-inactivated Mb group and BCG group) and unvaccinated badgers, macrophages existed extensively in the granulomas, while B cells and plasma cells were less in abundance. And T cells were not found in above granulomas. These results illustrated that phagocytic cells were prone to promote non-specific innate responses rather than adaptative humoral immune responses. The plasma cells of the heat-inactivated Mb group were more than those of the BCG group, which indicated the formation of adaptative humoral responses (116).

Additionally, heat-inactivated vaccine was used to vaccinate in zebrafish model for controlling fish mycobacteriosis. In the Mycobacterium marinum challenged zebrafish model, intraperitoneal injection of heat-inactivated Mb could significantly promote the survival rate (30–70%), and reduce the content of mycobacteria in granulomas by detecting the DNA level of Mycobacterium marinum. Through mycobacteria staining and immunolabelling, the qualified granulomas in zebrafish of the heat-inactivated vaccine group were apparently less than those of the unvaccinated group. To identify the influence of vaccination on immune reaction, the mRNA levels of akr2, C3 and IL-1β were evaluated by qRT-PCR. And the results demonstrated a C3 pathway-based innate immune response with the regulation of Ark2 protein, which was consistent with the protective mechanisms in other species (117). Furthermore, mucosal administration of the heat-inactivated Mb by immersion was conducted in zebrafish. And it provided a similar protection effects in the vaccinated group against Mycobacterium marinum, which stimulated IgM antibodies for Mb antigens. The C3 pathway-based innate immune response was found to be the protective mechanisms as well (118).

Due to the complicated relationship behind tuberculosis infection among different livestock and wild animals, the strain sources of heat-inactivated vaccines are expansive. Therefore, the chosen strains are not only related to protection efficacy, but also important to prevent subsequent spreading of antigenicity factor to other animal species, which may interfere with the results of tuberculosis diagnosis. Considering the living habits and immune characteristics of diverse wild animals, it is hard to set a standard study protocol suitable for various hosts. Unlike vaccination studies for human and livestock, the heat-inactivated vaccines can be prepared as baits for once or multiple oral administration, which is much more feasible in practical world. Hence, it provides more possibilities to make further progress in developing reliable inactivated vaccines according to the immune characteristics of wildlife.

## Live attenuated vaccines

Different from classic vaccines, the immunogenicity of live attenuated vaccines is acquired from treated pathogens with weakened toxicity, which achieves a balance between immune response and non-pathogenicity. In recent years, the research on live attenuate vaccines against human and animal tuberculosis is quite active.

### Mtb mutants

Levillain et al. studied a live attenuated vaccine on the basis of GC1237 mutant which caused the outbreak of tuberculosis on Canary Island. The mutant strain was inactivated both in Rv1503c gene (responsible for surface glycolipid synthesis) and two-component global regulator PhoPR. This vaccine retained obvious virulence in the intravenous infection model of immunodeficient SCID mice. Therefore, researchers attenuated the mutant again and eliminated the two-component system PhoPR which regulated the virulence of numerous Mtb by gene knockout. The latter vaccine had the same protective effect as BCG vaccine in CB6F1 mice and C3H/HeNRj mice. Additionally, considering that the vaccine originated from the Beijing strain, C57BL/6 mice were challenged with Beijing strain HN878 and European and American strain M2 respectively. And it was found that the vaccine had stronger resistance to HN878 than M2 as expected (119). The results indicated that live attenuated vaccines were prone to be influenced by genetic background, which might generate BCG vaccines with diverse protective power around the world. It also suggested that local vaccines could be prepared from strains prevalent in corresponding regions.

ST28 is a mutant of Mtb, which lacks the gene encoding the extracellular sigma factor SigE. It is strongly attenuated in comparison with BCG vaccine. But ST28 can induce more effective protection of mice from Mtb infection. After inoculation of guinea pigs, the CFU formation and lung lesion area challenged by the highly virulent tuberculosis strain H37Rv were reduced, and the protective effect was similar to that of BCG vaccine. However, its protection mechanism is still not clear (120).

Rogelio-Hernandez-Pando et al. constructed and identified a label-free double mutant of Mtb sigE fadD26, which was also weaker than BCG vaccine, and more effective than single sigE mutant. Its protective effect was similar to that of BCG vaccine in guinea pig model. The results indicated that sigE fadD26 mutant might be used as an effective candidate to replace or supplement BCG vaccine (121). MTBVAC vaccine was also a promising alternative to BCG vaccine, which was based on the modified form of human Mtb gene. The vaccine was relatively safe with immunogenicity to tuberculosis antigens of adults and newborns. By inducing glycolysis, glutamine decomposition and accumulating histone methylation markers at the promoter of pro-inflammatory genes, MTBVAC vaccine could produce trained immunity as well. Trained immunity is related to the long-term epigenetic and metabolic reprogramming of innate immune system cells. Therefore, if it has a better protective effect on tuberculosis in the third stage efficacy test, this vaccine may be considered as a substitute for BCG vaccine available at present (122). Although it has been a long time since the invention of BCG vaccine, which represents the most successful Mtb mutant, there is no breakthrough in exploring novel alternatives. The targeted genes are involved in virulence but do not participate in immune protection. Nevertheless, the limited knowledge about the specific mechanism of host-Mtb interactions and related genes accounting for immune response hinders the development of ideal mutant candidates. Until now, researchers are still seeking genomic loci eliminating virulence from Mtb genome without damaging immunogenicity of candidate vaccine strains. This dilemma may continue until figuring out the relationship between Mtb genome and immune effect. And the situation of subsequent mentioned Mb mutant is similar.

## Mb mutants

The knockout of causative genes from wild type mycobacterium pathogens is an appealing approach for circumventing BCG's insufficient protection. Accordingly, in order to seek potential candidate vaccines for bovine tuberculosis, Federico Carlos Blanco et al. generated an attenuated mutant strain (Δmce2) of Mb by deleting mce2A and mce2B genes, and detected its immunological properties in a cattle model of bovine tuberculosis. The cattle administrated with Δmce2 exhibited an equivalent activation of CD4^+^ T cells to that of cattle infected with the original Mb strain. And the amount of mRNA Th1 cytokines was higher in the peripheral blood of the Δmce2 group after *in vitro* stimulation. Moreover, in comparison with the BCG group, the histopathological lesion scores of the Δmce2 group were notably lower. However, the inoculation of Δmce2 in cattle resulted in a positive response of tuberculin skin test, which might be further modified by locating and eliminating related immunogenic genes. And this Δmce2 was considered to be a promising vaccine for cattle against Mb (123, 124).

As revealed in a previous review, phoP gene involves in the adjustment of Mb lipid metabolism, which is associated with down-regulated expression of diacyltrehaloses, polyacyltrehaloses and sulfolipids. Additionally, phoP gene affects Mtb complex virulence by influencing ESX-1 secretion system related genes as well. And the expression of ESAT6 and CFP10 in Mtb H37Rv ΔphoP and Mtb H37Ra is obviously decreased (125). Hence, Elizabeth and collaborators knocked out phoP gene (Rv0757) in Δmce2 to obtain a novel Mb mutant strain (Δmce2-phoP). It was observed that the virulence of Δmce2-phoP was significantly attenuated than that of wild type strain in mice. By compared with the BCG group, Δmce2-phoP inoculation stimulated Th1 cytokines after 30 days in mice without statistical differences. And the protection of Δmce2-phoP was proved to be a potential candidate as Mb vaccine in cattle (126).

Together with p55, which encodes an efflux pump or transporter, the gene that encodes P27/LprG forms a virulence operon. On this basis, a study constructed an Mb mutant Δp27-p55, which exhibited a distinct attenuation than its original strain, but more infectious than BCG Pasteur in nude mice. Mb or Mtb strains challenged mice and guinea pigs were used to evaluate the protection of Δp27-p55. The results of pathology and bacterial loads in spleen were similar between Δp27-p55 and BCG vaccine, while the effect of Δp27-p55 were inferior to that of BCG vaccine in lung. However, the lower amount of IFN-γ and IL-2 negative expression in cattle of the Δp27-p55 group represented an inadequate Th1 response, which indicating P27's immunogenic potency. And further research need to be conducted to improve Δp27-p55 as a TB vaccine (51, 127).

Although the tests of Differentiating Infected from Vaccinated Animals (DIVA) for tuberculosis have been invented, their cost is high, accompanying with an insufficient sensitivity. Hence, vaccines without disturbance to animal tuberculosis detection are significant for clinical practice. This dilemma may be solved by means of searching potential DIVA antigens from Mb or locating the antigens irrelevant to the persistence and defense in BCG vaccine. According to this clue, a diagnostic-compatible triple knock-out ΔBCG TK strain was reported by deleting unnecessary genes in BCG Danish, which showed a similar protection effect to wild-type BCG in Mb-infected guinea pigs (51). In contrast to other potential tuberculosis vaccines other than BCG vaccine, this study provides a possibility of generating modified BCG vaccines not only keep protection potency against Mb infection, but also satisfy the DIVA skin test for controlling animal tuberculosis in future.

## Subunit vaccines

The deficiency of viral nucleic acid ensures the safety of subunit vaccines, which contain typical proteins isolated from pathogens. And the effect of this vaccine depends largely on the selected antigen proteins (128). Tuberculosis-related antigens are summarized in Table 2. For tuberculosis, the antigen Acr1 and TB10.4 of incubation period and active period were chosen respectively to construct the multi-stage double epitope vaccine L4.8 (129). By reducing the bacterial load, and increasing the number of CD4^+^ T cells and CD8^+^ T cells in mice, L4.8 exhibited superior protective effect than that of BCG vaccine. In addition, Kim et al. studied the InsB antigen, an ESAT-6-like antigen which belonged to the Mtb9.9 subfamily of the Esx family. The results showed that InsB might be an excellent vaccine antigen component, which was conducive to develop a multi-antigen Mtb subunit vaccine by producing Th1 biased memory T cells with multifunction ability. And its anti-tuberculosis protection was similar to that of ESAT-6. More importantly, the vaccine might have long-lasting protection against high virulence Mtb K (130).

**Table 2:** Summary of tuberculosis antigens.

**Antigen**	**Mode of action**	**Validation animal (delivery route) and** **cell**	**References**
Acr1	Induce the production of interferon- γ	Female BALB/c mice (SC), spleen cells	Rai et al. (129)
TB10.4	CD8^+^ T cell epitope of the active form of Mtb	Female BALB/c mice (SC), spleen cells	Rai et al. (129)
InsB	Improve the sensitivity of human and mouse immunodiagnosis	Female C57BL/6 mice (SC), splenocyte and lung cells	Kim et al. (130)
CFP-10	Plays a key role in the virulence of Mycobacterium tuberculosis	Female C57BL/6 mice (SC), Escherichia coli TOP10F'	Baghani et al. (131)
Ag85b	Provide strong immunogenicity	Female C57BL/6 mice (IM), peripheral blood mononuclear cells	Counoupas et al. (91)
Ag85a		Human volunteers (SC)	Vasina et al. (80)
ESAT-6	Elicits strong T cell recall immune responses	Female C57BL/6 mice (SC), spleen and lung cells	Choi et al. (132)
Rv2299c	Induce DC maturation	Female C57BL/6 mice, OT-I and OT-II T-cell receptor	Choi et al. (69)
		Transgenic mice, C57BL/6, C57BL/6J TLR2 knockout mice, andC57BL/10 TLR4 knockout mice (SC), murine bone	
		Marrow-derived DCs	
HSP90	Activate TLR4 route	Female C57BL/6J mice (SC), murine bone marrow-derived DCs	Choi et al. (69)
HSP X	Induce the production of IFN-γ and IL-12	Female BALB/c mice (SC), spleen cells	Mansury et al. (70)
PPE44	Induce the production of pro-inflammatory factors and activate macrophages	Female BALB/c mice (SC), spleen cells	Mansury et al. (70)
EsxV	Considered as specific T cell targets	Female BALB/c mice (SC), spleen cells	Mansury et al. (70)

### Improvement of targeting performance

Accurate positioning is vital to prevent and control kinds of diseases including tuberculosis, which not only reduces medication dosage and side effect, but also enhances therapeutic effect and patient compliance. For a better prevention efficiency of subunit vaccines, bioactive ligands including Fc domain and APC surface molecule have been utilized to target tuberculosis immune system. Baghani et al. developed a subunit vaccine consisting of Mtb CFP-10 and the Fc domain of mouse IgG2a which could target APC. Further evaluation in mice showed that this vaccine induced a better Th1-type immune response than the control groups (BCG vaccine and PBS). It implies that subunit vaccines based on the fusion immunogens containing distinct functional domains are potential candidates with ability to target vital immune cells (131). In addition to antibodies, some APC surface molecules can also be utilized to realize targeting. Anneliese S. Tyne et al. used Mtb antigen cutinase-like protein (Culp) 1–6 and MPT83 directly combining with a new adjuvant Lipokel (Lipotek Pty Ltd.) to construct a tuberculosis subunit vaccine. Lipokel is a ligand for TLR2, which allows the vaccine to target TLR2-expressing immune cells (monocytes, macrophages, dendritic cells, and so on). Seven days after the mice were inoculated, the Culp1-6-Lipokel group had significantly increased lung neutrophils, myeloid DCs, recruited monocytes and alveolar macrophages compared with the simple Lipokel group. Both the Culp1-6-Lipokel group and the MPT83-Lipokel group showed an increase in the number of activated DCs, but only the MPT83-Lipokel group lasted until 28 days. It was encouraging that CD4^+^ T cells in the MPT83-Lipokel group increased significantly after 28 days of vaccination (133). Besides immunological activity, the size and connection pattern are noteworthy features of target components, which may affect the original immunogenicity of subunit vaccine. Moreover, there is rarely reported that common target molecules used for drug delivery are applied to improve the targeting performance of targeting performances. Considering various types of these target molecules, it may be a potential way to create more ideal subunit vaccines with superior anti-tuberculosis effects.

### Assistance of adjuvants

Due to the relatively low immunogenicity of subunit vaccines, it is necessary to employ adjuvants for enhancing immune efficiency. And it has been proved that the immune potency was improved obviously by introducing appropriate adjuvants (87). At present, most of these adjuvants are involved in Th1-type reaction derived from CD4^+^ T cells, which plays an important role in anti-tuberculosis immunity.

The Ag85B and CysD antigen protein fusion mucosal vaccine used deltamethrin Advax as an adjuvant, and its performance by intratracheal administration was better than that by parenteral administration. The effect of Advax mainly depends on the CD4^+^ T cells residing in lungs which produce IL-17 and retinoid-related orphan nuclear receptor γt (RORγt) (91). Among them, RORγt is involved in the development of Th17 cells, which further support Th1 response and accelerate the clearance of pathogens (134, 135). In another study, Advax adjuvant was applied to develop an anthrax subunit vaccine with recombinant protective antigen. After the inoculation and challenge experiments within mice, the vaccine exhibited good protective efficacy against Bacillus anthracis 7702 strain with a relatively high safety (136).

Retinoic acid (RA), a metabolite of vitamin A, can regulate the differentiation of CD4^+^ T cells, and subsequently affect the homeostasis of dendritic cells and the homing of T cells and B cells (137–139). Mice immunized by subcutaneous injection of tuberculosis subunit vaccine (CAF01 + H56) in the presence of RA showed intensive mucosal H56 specific IgA response and ameliorative Ag-specific CD4^+^ T lymphocyte homing to lung (92). Hence, RA can be considered in the development of tuberculosis mucosal vaccine. Furthermore, Pro-Glu/Pro-Pro-Glu (PE/PEE) family proteins are significant in the pathogenesis of tuberculosis. Started with CD4^+^ T cells in the same way as RA, researchers fused ESAT-6 with the common CD4^+^ T cell epitope of PE/PEE family. After inoculation of mice, CD4^+^ T cells secreting IL-2 and IFN-γ were induced, and the protective effect on mice was strengthened (132). It manifests that the optimization of essential epitopes with satisfactory immunogenicity is a promising strategy for exploring alternatives of complicated antigens.

Except the adaptive immune response associated with CD4^+^ T cells, the innate immune response also contributes to protecting against tuberculosis infection. α-galactosylceramide not only stimulates T cells to produce antigen-specific IFN-γ-mediated Th1 immunity fighting against tuberculosis, but also activates natural killer cells in innate immunity (140, 141). After combined with α-galactosylceramide, the immune effect of Mtb antigens (Ag85B and ESAT-6) was raised by more than 50 times (93). Meanwhile, the efficiency of the Ag85B-ESAT6 fusion subunit vaccine was also increased through utilizing DC-activated antigen, Rv2299c, as an adjuvant (69, 142).

Nanoemulsion (NE) is a kind of oil in water emulsion, which can be prepared with antigens. It has a nice assistant effect when combined with influenza vaccine (143). What's more, NE together with Mtb specific antigen could elicit an obvious mucosal IL-17 T cell response (94). Additionally, CAF01 is a compound based on dimethyl octadecyl ammonium bromide and trehalose-6,60-dibenzoate, which promote antigen presentation to dendritic cells and activate dendritic cells (144). CAF01 can be made into powder by freeze-drying method, removing the cold-chain transportation restriction of vaccines. And the freeze-dried H56 tuberculosis subunit vaccine was equivalent to the unfreeze-dried vaccine in anti-tuberculosis, which induced antigen-specific Th1, Th17 and humoral immune responses as well (95). These freeze-dried vaccines reduce transportation cost and guarantee stability, which will be of great benefit to tuberculosis prevention and treatment in economically undeveloped area.

### Utilization of immunoinformatic tools

As the derivative of big data and artificial intelligence (AI) technology, bioinformatics tools can also be utilized to screen effective antigen epitopes for fabricating potential vaccine candidates for tuberculosis (145). Owing to the important function of DNA binding proteins in the process of DNA replication, transcription, regulation and repair, Sunita et al. applied DNABIND tool to identify 1453 DNA binding proteins from the genes of Mtb for screening potential tuberculosis vaccines. Through ABCpred server, 18 DNA binding proteins were chosen to analyze the B-cell epitopes. And antigenic and non-allergenic B-cell epitopes were decided to predict the T-cell epitopes by ProPredI and ProPred server. Then, the evaluated T-cell epitopes (Rv1088, Rv3923c, Rv3235, Rv2871, Rv2731 and Rv0707) and DNA binding proteins were used for structural modeling to determine the location within the corresponding proteins. And the interaction of these epitope and human leukocyte antigens were finally detected to prove their effects, which implied their potential as vaccine candidates against tuberculosis (146). Similarly, enhanced intracellular survival proteins from Mtb were exploited to construct a chimeric vaccine by immunoinformatics approaches. The combined results of toxicity, allergenicity, antigenicity, MHC allele binding and IFN epitopes predicted 8 cytotoxic T lymphocyte epitopes, 6 helper T lymphocyte epitopes and a B cell epitope as suitable construction components. Further structure modification and 3D structure analysis of the chimeric vaccine were utilized to validate docking studies with Toll-like receptor 4. And their stable interaction was proved by molecular dynamic simulation, which provided a chimeric vaccine candidate for subsequent *in vitro* and *in vivo* test (147). The frequently-used bioinformatic tools and corresponding applications are listed in Table 3. The application of immunoinformatic tools, which provides numerous reliable combinations of different epitopes, facilitates the construction of tuberculosis vaccines. Meanwhile, it simplifies the preliminary screening process of the obtained outcomes without practical operation (7, 20, 21). Moreover, the completion of genome sequencing of Mtb, the development of computer technology, and the excellent safety of subunit vaccine may be the reasons why tuberculosis subunit vaccines have attracted more attention in recent years (148, 149). At the same time, further development and intersection of bioinformatics, genomics, proteomics and system biology has provided more opportunities for the evolution of subunit vaccines (150).

**Table 3:** Summary of common immunoinformatic tools.

**Tool**	**Function**
DNABIND	Identify the target gene sequence of Mtb strains
VaxiJen v2.0	Assess the antigen characteristic of target gene; Analysis the antigenicity of predicted epitopes
AllerTOP v2.0	Screen the allergen free protein sequences in nature
ABCpred	Predict the B cell epitope of protein
ProPred	Predict the T cell epitope interacting with MHC class I and class II molecules
PDBsum	Predict the secondary structure of protein
SWISS-MODEL	Predict the three-dimensional model of protein
PEP-FOLD	Predict the three-dimensional structure of protein; Simulate protein docking
FRODOCK v2.0	Predict the docking between human leukocyte antigen and protein
GRAMM-X Protein-Protein Docking Web Server v1.2.0	Simulate proteins and estimate their immunity

## DNA vaccines

DNA vaccines are recombinant eukaryotic expression vectors encoding antigens. After administration, the exogenous gene is expressed *in vivo*. The resulting antigens activate the body's immune system, thus inducing specific humoral immunity and cellular immune response. According to this principle, the safety of DNA vaccine is excellent, which is similar to subunit vaccines. The key to both vaccines lies in the selection of antigen, but DNA vaccine focuses on antigen gene. For example, Mce1A gene may be involved in the invasion and survival of Mtb in human macrophages (151, 152). It is suggested that the selection of antigen genes should start from the pathogenesis of tuberculosis and block the pathogenic pathway of Mtb. Some genes employed by DNA vaccines against tuberculosis are shown in Table 4.

**Table 4:** Summary of genes employed by DNA vaccines against tuberculosis.

**Gene**	**Mode of action**	**Validation animal (delivery route) and** **cell**	**Reference**
C6	Activate immune pathways against tuberculosis of T cells	BALB/c and C57BL/6 female mice (IM), CHO cells	Maurya et al. (153)
HAHB	Improve the effect of BCG	Female BALB/c mice (IM), HeLa cells	Teimourpour et al. (154)
Mtb32C	Enhance CD8^+^ T cell-dependent protective immunity and induce higher IFN-γ production	Female BALB/c mice (IM), HeLa cells	Teimourpour et al. (154)
Rv2029c, Rv2031c, Rv2627c	Induce strong T cell immune responses	preliminary computational analysis	Moradi et al. (155)
Ag85A	Involved in the biogenesis of mycobacterium cell wall	Female BALB/c mice (IM), spleen cells	Peeridogaheh et al. (2)
CFP-10	Activate T cells	Female BALB/c mice (IM), spleen cells	Baghani et al. (131)
HSP65	Induce cellular immune response and stimulate the production of IFN-γ and Cytotoxic T lymphocyte	Cynomolgus monkeys, 15K granulysin Tg mice, 3 types of 9K granulysin Tg mice (IM), cells of lung, liver and spleen	Okada et al. (156)

Considering that BCG vaccine could not eliminate latent Mtb infection (157), some DNA vaccines were designed complying with prime-boost strategy. C6 vaccine contains DNA sequence of six immunodominant CD4^+^ and CD8^+^ T cell epitopes of Mtb expressed in latent, acute and chronic infection stages, respectively. After inoculation of mice, the release of IFN-γ and TNF-α grew, and the activation of dendritic cells and macrophages increased. The bacterial load in lung and spleen of the C6 + BCG vaccine group mice was significantly reduced (153). Moreover, prime-boost strategy shows greater advantages compared with the DNA vaccine alone. Teimourpour et al. constructed the DNA plasmid vaccine encoding Mtb32C-HBHA fusion protein, which was verified by RT-PCR and Western blot *in vitro*. When the mice were inoculated with the immunization strategy promoted by DNA vaccine and primary immunization with BCG vaccine, the yield of IFN-γ was superior to that of the BCG group and the DNA vaccine group alone. In addition, a large amount of IL-12 and TGF-β were also induced successfully, which was sufficient to effectively stimulate the organism immune system (154).

Other than above prime-boost strategy, the application of immunoinformatics tools to exploit DNA vaccines can also be used as supplementary. Moradi et al. designed a candidate DNA vaccine consisting of three tuberculosis latency antigens Rv2029c, Rv2031c, and Rv2627c, along with microtubule-associated protein light chain 3 (LC3). It is expected that LC3 can increase the reaction of CD4^+^ T cells through autophagy. It has been reported that autophagy promotes the recognition and processing of MHC-2 molecules to antigens (158, 159). Moreover, there are 8 methods for predicting MHC class I-binding peptides: Artificial neural network (ANN); average relative binding (ARB); stabilized matrix method (SMM); stabilized matrix method with a peptide: MHC binding energy covariance matrix (SMMPMBEC); scoring matrices derived from combinatorial peptide libraries (Comblib-Sidney2008); Consensus; NetMHCpan and IEDB. After preliminary computational analysis, the vaccines can be used as candidate vaccines against tuberculosis, but it still needs animal experiments to prove its ability (155).

Although the construction technology of DNA vaccines is relatively mature and convenient, its immune potency needs further optimization. Peeridogaheh et al. constructed Ag85a-CFP10 fusion DNA vaccine, and the RT-PCR results illuminated the introduction of target mRNA in eukaryotic cells. Compared with the BCG group, vaccinated mice produced more IL-4, IFN-γ and TGF-β, while the level of IL-12 did not rise, which indicated that the vaccine cannot potentially induce cellular immunity (2).

### Enhancement of immunogenicity

As a newly developed product, DNA vaccines possess some distinct advantages, such as no need to express and purify antigens *in vitro*, easy preparation and low cost. However, its immunogenicity and physiologic stability are slightly poor (160).

On one hand, some special gene sequences encoding substances that promote anti-tuberculosis immunity can be utilized as adjuvants of DNA vaccines by constructing them into a plasmid together with various antigen genes. For example, FMS-like tyrosine kinase 3 plays an important part in the proliferation and differentiation of myeloid and lymphoid progenitor cells and dendritic cells (DCs) (161). As a promising adjuvant, FMS-like tyrosine kinase 3 can enhance the recruitment and amplification of DC, and facilitate antigen uptake and cross-presentation (162, 163). Ganzhu Feng et al. developed a recombinant DNA vaccine based on nanoparticles, which contained three T cell epitopes of ESAT-6 and FMS-like tyrosine kinase 3 gene, and was encapsulated by chitosan (CS) nanoparticles. The results showed that the vaccine significantly enhanced T cell immunity and protected against Mtb H37Rv attack (22, 164).

On the other hand, some non-viral vector materials are used as adjuvants, which can protect plasmids from degradation, improve transfection efficiency and enhance the immunogenicity of DNA vaccines. Moreover, these materials (liposomes, polylactic acid-glycolic acid copolymer, polyethylene glycol, etc.) can be further modified to target specific immune cells (165, 166). Rosada et al. developed cationic liposome-based HSP65 DNA vaccines with construction either by encapsulation or adsorption, and compared the difference between intramuscular injection and intranasal administration. The study found both liposome preparations induced a typical Th1-type immune response pattern, but the intramuscular delivery pathway did not reduce the number of bacteria. However, a single intranasal immunization with HSP65 complexes carried plasmid DNA as low as 25 μg resulted in a significant quantity reduction of bacterium in lungs. These effects were accompanied by increasing IFN-γ level and preserving the lung parenchyma, which were similar to those found in mice given four intramuscular injections of naked DNA-HSP65 (167). Furthermore, the application of nanotechnology can improve the effectiveness of vaccines (168). Maytal Bivas-Benita et al. coated a DNA plasmid encoding eight HLA-A^*^0201 restricted T cell epitopes of Mtb with chitosan. By comparing with common intramuscular immune route, pulmonary administration of the DNA plasmid incorporating chitosan nanoparticles raised the secretion level of IFN-γ. And the immunogenicity was improved by the chitosan nanoparticles as well (97). It reveals the potential influence of vaccination route on vaccine efficacy. Considering the characteristics of different non-viral vectors, novel inoculation routes such as pulmonary, transdermal and nasal administration, are encouraged to take attempts in future vaccine design and clinical trials.

### Application of viral vectors

Apart from non-viral vectors, viral vectors have also been studied for DNA vaccines. Okada et al. developed a DNA vaccine expressing tuberculosis antigen HSP65 and cytokine IL-12, which was encapsulated by Japanese hemagglutination virus (HVJ). The study found that the ability to induce IFN-γ and IL-12 is stronger when using 100 μg DNA, which was better than the PDD control group (156). Mangalakumari Jeyanathan et al. developed an adenovirus-based tuberculosis vaccine for respiratory inoculation, which provided a valuable reference of aerosol vaccine strategies. And it was also expected to be transformed into a vaccine against Corona Virus Disease 2019 (COVID-19) (169). However, viral vectors have some inherent disadvantages, for example, small encapsulation size and security threat caused by integration into host genome (170). In consideration of safety, non-viral vector candidates with modified immunity will be promising DNA vaccine adjuvants for large-scale clinical application in future.

## Conclusions and perspectives

Tuberculosis is still one of the most important infectious diseases in human history because of its unique characteristics. As prevention can solve tuberculosis fundamentally, the development of vaccines is a very important job. Until now, there have been numerous candidate vaccines for anti-tuberculosis, but few of them have successfully applied to clinical practice. This situation warns people of the urgency to develop novel tuberculosis vaccines. Otherwise, the goal of reducing the incidence and mortality of tuberculosis by 90 and 95% respectively before 2035 may become hard to achieve (171).

Fortunately, there have been many studies concentrated on subunit vaccines against tuberculosis in recent years. As mentioned above, targeting and immunogenicity improvement have been achieved under laboratory conditions. Subunit vaccines have also appeared more frequently than other types of tuberculosis vaccines in clinical trials in the past decade, which indicates their potential for practical therapeutic application. The clinical trials of tuberculosis vaccines in recent years are shown in Table 5. Subunit vaccine and DNA vaccine are both new generation vaccines with several similarities, such as high safety, multiple antigen mechanism and relatively low immunogenicity. Introducing targeting and adjuvants for subunit vaccines are also applicable to DNA vaccines. Nevertheless, the implemented strategy is different between protein and plasmid. For example, Ali Asghar Baghani et al. fabricated the fusion protein of tuberculosis antigen CFP-10 and Fc domain of mouse IgG2a to achieve targeting. While the DNA vaccine can package CFP-10 encoding plasmid with a carrier, and then connect the Fc domain of mouse IgG2a with the carrier through covalent bond or electrostatic interaction to achieve targeting. What is noteworthy is that tuberculosis is a poverty-related disease, which reminds us to pay more attention to controlling the cost (172). From this point of view, DNA vaccine is more economical than subunit vaccine, which does not need protein purification, but directly immunizes with plasmids.

**Table 5:** The clinical trials of TB vaccines in recent years.

**Type of** **vaccine**	**Vaccine name**	**Vaccine composition**	**Stage**	**Start date**	**Completion** **date**	**Status**	**ClinicalTrials.gov** **Identifier**
Subunit vaccine	AEC/BC02	Ag85B, ESAT-6, CFP-10 and complex adjuvant system BC02	I	2020.05	2022.04	Active, not recruiting	NCT04239313
			II	2022.01	2023.10 ^*^	Recruiting	NCT05284812
			I	2018.04	2019.10	Completed	NCT03026972
	GamTBvac	ESAT-6, CFP-10, Ag85A, adjuvant dextran and CpG oligonucleotide	I	2017.01	2017.12.13	Completed	NCT03255278
			III	2022.01	2025.11^*^	Recruiting	NCT04975737
			II	2018.12	2020.05	Completed	NCT03878004
	GSK692342	Recombinant fusion protein (Mtb72f) and GlaxoSmithKline adjuvant system	II	2014.08	2018.1	Completed	NCT01755598
			II	2012.08	2013.05	Completed	NCT01669096
	H56:IC31	Ag85B, ESAT-6, Rv2660c	I	2015	2020.03	Completed	NCT02503839
			II	2019.01	2024.12 ^*^	Recruiting	NCT03512249
			I	2015.09	2016.12	Completed	NCT02378207
			I/II	2013.08	2015.12	Completed	NCT01865487
			I	2014.11	2016.10	Completed	NCT02375698
			II	2018.06^*^	2021.06^*^	Withdrawn	NCT03265977
	H4:IC31	Ag85B, TB10.4 and TLR9 agonist adjuvant IC31^®^	I	2015.09	2016.12	Completed	NCT02378207
			I/II	2013.07.01	2017.12	Completed	NCT01861730
			II	2014.02	2017.10	Completed	NCT02075203
	ID93+GLA-SE	Rv2608, Rv3619, and Rv3620, latency antigen Rv1813 and GLA-SE adjuvant.	II	2018.05.31	2020.06	Unknown	NCT03806686
			II a	2015.06	2017.01	Completed	NCT02465216
			I	2019.04	2020.12^*^	Unknown	NCT03806699
			I	2013.09	2015.07	Completed	NCT01927159
			I	2012.08	2014.05	Completed	NCT01599897
			I	2018.10	2020.06	Completed	NCT03722472
			I	2015.10	217.08	Completed	NCT02508376
	M72/AS01E	Mtb39A, Mtb32A and adjuvant system AS01E	II	2020.11	2022.08^*^	Active, not recruiting	NCT04556981
Viral vector vaccine	TB/FLU-01L	ESAT-6	I	2013.10	2015.02	Completed	NCT03017378
			I	2013.10	2015.02	Completed	NCT02501421
	MVA85A	Ag85A	I	2013.10	2016.01	Completed	NCT01954563
			I	2015.09	2018.10	Terminated	NCT02532036
			I/II	2015.09	2018.03	Completed	NCT02729571
			I	2013.01	2014.11	Completed	NCT02013245
			II	2014.06	2015.01	Completed	NCT02178748
			II	2012.10	2015.05	Completed	NCT01650389
			I/II	2018.05	2021.09	Completed	NCT02933281
			I	2013.07	2014.12	Completed	NCT01879163
			I	2012.09	2014.08	Completed	NCT01683773
			III	2022.07^*^	2029.09^*^	Not yet recruiting	NCT04975178
			II	2019.02	2022.03^*^	Active, not recruiting	NCT03536117
			I/II	2019.07	2022.01^*^	Active, not recruiting	NCT03681860
	Ad5Ag85A	Ag85A	I	2017.09	2021.09	Completed	NCT02337270
	AERAS-402	Ag85a, Ag85b, TB10.4	I	2012.09	2014.08	Completed	NCT01683773
	ChAdOx185A	Ag85A	I	2013.07	2016.04	Completed	NCT01829490
			I	2019.01	2020.08	Completed	NCT04121494
			I/II	2019.07	2022.01^*^	Active, not recruiting	NCT03681860
Inactivated vaccine	RUTI	Mycobacterium tuberculosis cell debris and liposome carriers	II	2021.09	2025.11^*^	Recruiting	NCT04919239
			II	2020.03	2021.09^*^	Recruiting	NCT02711735
			II	2021.12	2022.12^*^	Not yet recruiting	NCT05136833
	DAR-901	Dar901 is a whole cell inactivated tuberculosis vaccine for booster after BCG vaccination.	II	2016.03	2020.02	Completed	NCT02712424
			I	2014.02	2016.06	Completed	NCT02063555
Recombinant BCG vaccine	VPM1002	Recombinant BCG inserted into the Listeria toxin O gene	II	2015.06	2017.11	Completed	NCT02391415
			III	2020.11	2025.11^*^	Recruiting	NCT04351685
DNA vaccine	GX-70	Four-antigen plasmids from MTB together with recombinant Flt3 ligand	I	2018.03^*^	2018.08^*^	Withdrawn	NCT03159975

* Estimated time.

In addition, the evaluation results of some live attenuated vaccines seem to be superior to those of subunit vaccines and DNA vaccines. But the preparation of live attenuated vaccines mostly depends on the mutant strains of Mtb. Gene mutation has the characteristics of randomness, hazardous risk, and non-orientation. Although various gene editing techniques such as CRISPR/Cas9, zinc finger nucleases (ZFNs), transcription activator-like effector nucleases (TALENs), and homing endonucleases have been established (173), we cannot guarantee feasible site-directed mutations for vaccines. Thus, exploring reliable mutation methods is crucial to screen potential live attenuated vaccines.

Consequently, the summary of current research progress of different tuberculosis vaccines has provided valuable experiences and suggestions, through which tuberculosis will finally be conquered in the near future.

## Author contributions

WQ: ideas and formulation or evolution of overarching research goals and aims, and revise the first draft. YG: preparation, creation and/or presentation of the published work, and specifically writing the initial draft (including substantive translation). YX: preparation, creation and/or presentation of the published work, and specifically revising the manuscript. JZ: preparation, creation and/or presentation of the published work, and specifically visualization/data presentation. ZW: oversight and leadership responsibility for the research activity planning and execution, including mentorship external to the core team. CD: performed the statistical analysis. YP: acquisition of the financial support for the project leading to this publication. All authors contributed to the article and approved the submitted version.

## Funding

This work was supported by National Key Research and Development Program of China (2017YFD0501400).

## Conflict of interest

The authors declare that the research was conducted in the absence of any commercial or financial relationships that could be construed as a potential conflict of interest.

## Publisher's note

All claims expressed in this article are solely those of the authors and do not necessarily represent those of their affiliated organizations, or those of the publisher, the editors and the reviewers. Any product that may be evaluated in this article, or claim that may be made by its manufacturer, is not guaranteed or endorsed by the publisher.
